# Experimental study on DEM parameters calibration for organic fertilizer by the particle swarm optimization − backpropagation neural networks

**DOI:** 10.1038/s41598-025-11827-9

**Published:** 2025-07-15

**Authors:** Fandi Zeng, Limin Liu, Yinzeng Liu, Hongbin Bai, Chunxiao Li, Zhihuan Zhao

**Affiliations:** 1 College of Mechanical and Electronic Engineering, Shandong Agriculture and Engineering University, Jinan, 250100 China; 2 College of Mechanical and Electrical Engineering, Inner Mongolia Agricultural University, Hohhot, 010018 China

**Keywords:** Organic fertilizer particles, Physical property, The discrete element model, Repose angle, The PSO − BP algorithm, Mechanical engineering, Mathematics and computing

## Abstract

In order to calibrate the properties of the organic fertilizer particles, this work employs an integrated strategy that combines simulations, machine vision techniques, and physical experiments. Through physical testing, the fundamental physical characteristics of the organic fertilizer particles were identified. The initial analysis was through the Plackett-Burman test. The parameters that greatly influence the angle of repose are established. The previously identified important variables were optimized by the Central Composite Design test. The regression fitting models of the BP neural network have been developed from the data set derived from the Central Composite Design test results. Genetic algorithms (GA) and particle swarm optimization algorithms (PSO) were used to optimize the BP neural network. The R^2^MAE and RMSE of the BP, GA − BP, PSO − BP and RSM regression models were compared and analyzed. The results showed that PSO − BP algorithm could achieve better fitting effect, and could construct a prediction model with higher accuracy and less error to analyze the repose angle of the organic fertilizer particles. The PSO − BP algorithm was used to iterate until the individual with the closest fitness was obtained. COR_O−p_ was 0.35, COS_O−O_ was 0.49, COS_O−p_ was 0.29 and COD_O−O_ was 0.38 were the optimal parameter combination.

## Introduction

Fertilizers made from organic materials, such as plant and animal waste, are referred to as organic fertilizers. Organic fertilizers, which are particularly well-suited for facility horticulture and organic growing systems, offer several advantages over chemical fertilizers in terms of enhancing soil structure, encouraging crop growth, safeguarding the environment, and increasing economic rewards^1–3^. The primary method of fertilization in our nation is now automated fertilization due to the quick advancement of horticulture fertilization technology and equipment. However, during mechanized fertilization operations, it is challenging for instruments and equipment to directly monitor the complicated inter-action forces between soil particles and fertilizer particles, as well as between organic fertilizer particles and fertilizer discharge mechanisms. Organic fertilizer particles are modeled using software based on the discrete element approach, and the motion state of the materials as well as the mechanism of interaction between the materials and mechanical devices are thoroughly and methodically investigated^4,5^. In addition to optimizing the mechanical device’s functioning and structural parameters, it can also increase mechanical performance and reduce expenses.

The calibration of discrete element properties of materials is fundamental for examining the interaction between materials and mechanical devices. Kruggel − Emden et al.^6^ studied the JKR model and proposed a method to improve the simulation efficiency. Grima et al.^7^ used the repose angle of particle pile in collapse test to calibrate the rolling friction factor required by the dry particles. Boac et al.^8^ used the discrete methods to simulate the material and interaction characteristics of selected oilseed particles. Wen et al.^9^ conducted the discrete element simulation of the granular fer-tilizer and calibrated the friction factor of the granular fertilizer. Yuan et al.^10^ carried out parameter calibration for the discrete element model of the organic fertilizer particles, and the relative error between the simulated repose angle and the actual repose angle was only 0.42%. Luo et al.^11^ calibrated the discrete element parameters of earthworm manure matrix based on the JKR bonding model, and the simulation results of the repose angle were close to the actual test results. Han et al.^12^ used a combination of the simulation and physical experiments to calibrate the discrete element simulation parameters of the loose manure in Xinjiang orchards. Gao et al.^13^ established a fertilizer block bonding model to explore the fertilizer crushing effect of the spiral groove wheel and its influence on the uniformity of fertilizer dis-charge. Chen et al.^14^ conducted an experimental study on sugarcane compound fertilizer with different moisture content, taking the repose angle as the response value. Shahrak et al.^15^ projected the adsorption capacity of water vapor in metal-organic framework (MOF) materials using two robust methods: artificial neural networks (ANN) and adaptive network-based fuzzy inference systems (ANFIS). Esfandyari et al.^16^ utilized two intelligent optimization techniques, namely artificial neural networks (ANNs) and adaptive neuro-fuzzy inference system (ANFIS), when combined with particle swarm optimization (PSO), to predict the heat transfer rate, Nusselt number, number of transfer units (NTU), and effectiveness of a double-pipe counter-flow heat exchanger. As machine learning technology advances, certain scholars employ genetic algorithms and neural networks to address the model, yielding more precise results^17–20^. The accuracy of the GA is evaluated against experimental data and a thermodynamic modeling technique utilizing the Peng-Robinson-Stryjek-Vera (PRSV) cubic equation of state, employing the Panagiotopoulos-Reid combining rule for this analysis^21^. In the conventional RSM approach, the efficacy of fitting may be influenced by the complexity of the problem and the size of the sample^22^. In contrast to typical multiple linear regression, the BP neural network possesses a robust capacity for fitting complex nonlinear functions^23–25^.

Currently, there is limited study on the parameter calibration of organic fertilizer particles utilizing the BP neural network. This article focuses on organic fertilizer as the subject of research, acquires the contact parameters of the organic fertilizer by physical testing, and develops a discrete element model. The regression fitting models of the BP neural network were created using the test results from the Central-Composite Design as the data set. The BP neural network was enhanced by the genetic algorithm (GA) and the particle swarm optimization method (PSO). The ideal combination of simulation parameters for organic fertilizer particles was achieved. This research aimed to accurately calibrate sheep manure organic fertilizer particles’ discrete element method (DEM) properties to enhance the simulation of how they behave during mechanized fertilization.

## Materials and methods

### Determination of test materials and intrinsic parameters

#### Determination of the basic parameters of the organic fertilizer particles

**Fig. 1:**
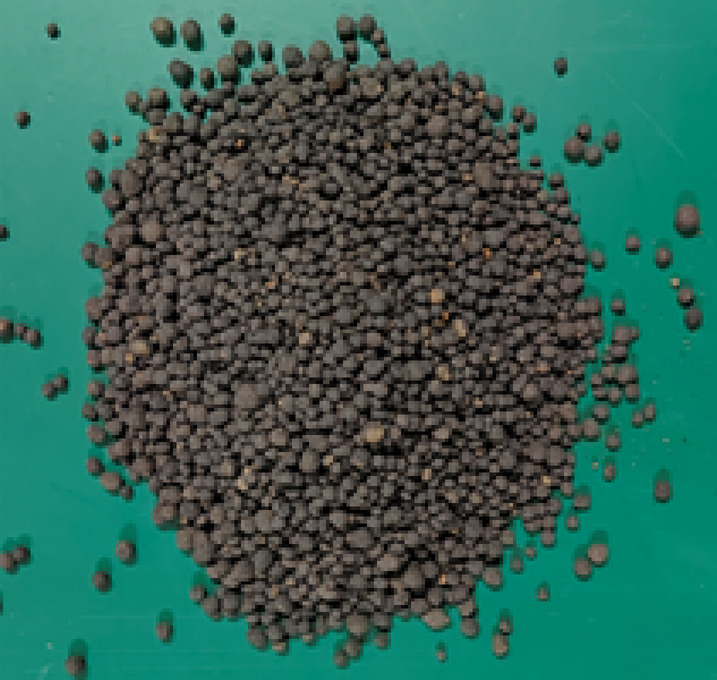
Organic fertilizer particles.

This paper selects the enduring sheep manure organic fertilizer manufactured by Inner Mongolia Pure Sheep Effective Organic Fertilizer Limited Liability Company, as depicted in Fig. 1. This organic fertilizer releases its fertility quickly after high-temperature fermentation, so it is suitable as a base fertilizer. The organic fertilizer enhances soil quality, stimulates root development, and improves plant luster, making it commonly utilized in vegetable growing. The density and moisture content of the organic fertilizer particles were assessed using the drainage and drying methods. Each group was replicated 10 times, the density of the organic fertilizer particles was 738 kg/m³, and the moisture content was 14.68%.

One hundred organic fertilizer particles were randomly picked, and each particle was measured using a vernier caliper. The distance along the major axis is the length (*L*_1_). The distance along the major axis is the width (*W*_1_). The vertical distance between the upper and lower surfaces is the thickness (*T*_1_). The dimensions *L*_1_, *W*_1_, and *T*_1_ of all particles were quantified. The equivalent diameter *D*_1_ and sphericity *φ*_1_ of the fertilizer particles are calculated, as shown in Formula (1).

1 $$\left\{ \begin{gathered} {D_1}=\sqrt[3]{{{L_1}{W_1}{T_1}}} \hfill \\ {\varphi _1}=\frac{{{D_1}}}{{{L_1}}} \times 100\% \hfill \\ \end{gathered} \right.$$

Where *D*_1_ is the equivalent diameter of the fertilizer particles, mm; *φ*_1_ is the sphericity of the fertilizer particles, %. *L*_1_ is the length of the fertilizer particles, mm. *W*_1_ is the width of the fertilizer particles, mm. *T*_1_ is the thickness of the fertilizer particles, mm.

Table 1 shows the form and size properties of pure sheep manure organic fertilizer particles. The dimensions of the organic fertilizer particles predominantly ranged from 6.75 mm to 3.13 mm. The width predominantly ranged from 3.12 to 5.44 mm. The thickness predominantly ranged from 2.94 to 5.65 mm. The corresponding diameter predominantly ranged from 3.12 to 5.48 mm. The sphericity predominantly ranged from 0.74 to 1.20 mm.

**Table 1:** The shape and size parameters of pure sheep manure organic fertilizer particles.

Parameters	Length L_1_(mm)	Width W_1_(mm)	Thickness T_1_(mm)	Equivalent diameter D_1_(mm)	Sphericity (%)
Maximum	6.75	5.44	5.65	5.48	1.20
Minimum	3.13	3.12	2.94	3.12	0.74
Average value	4.49	4.28	4.24	4.32	0.97

**Fig. 2:**
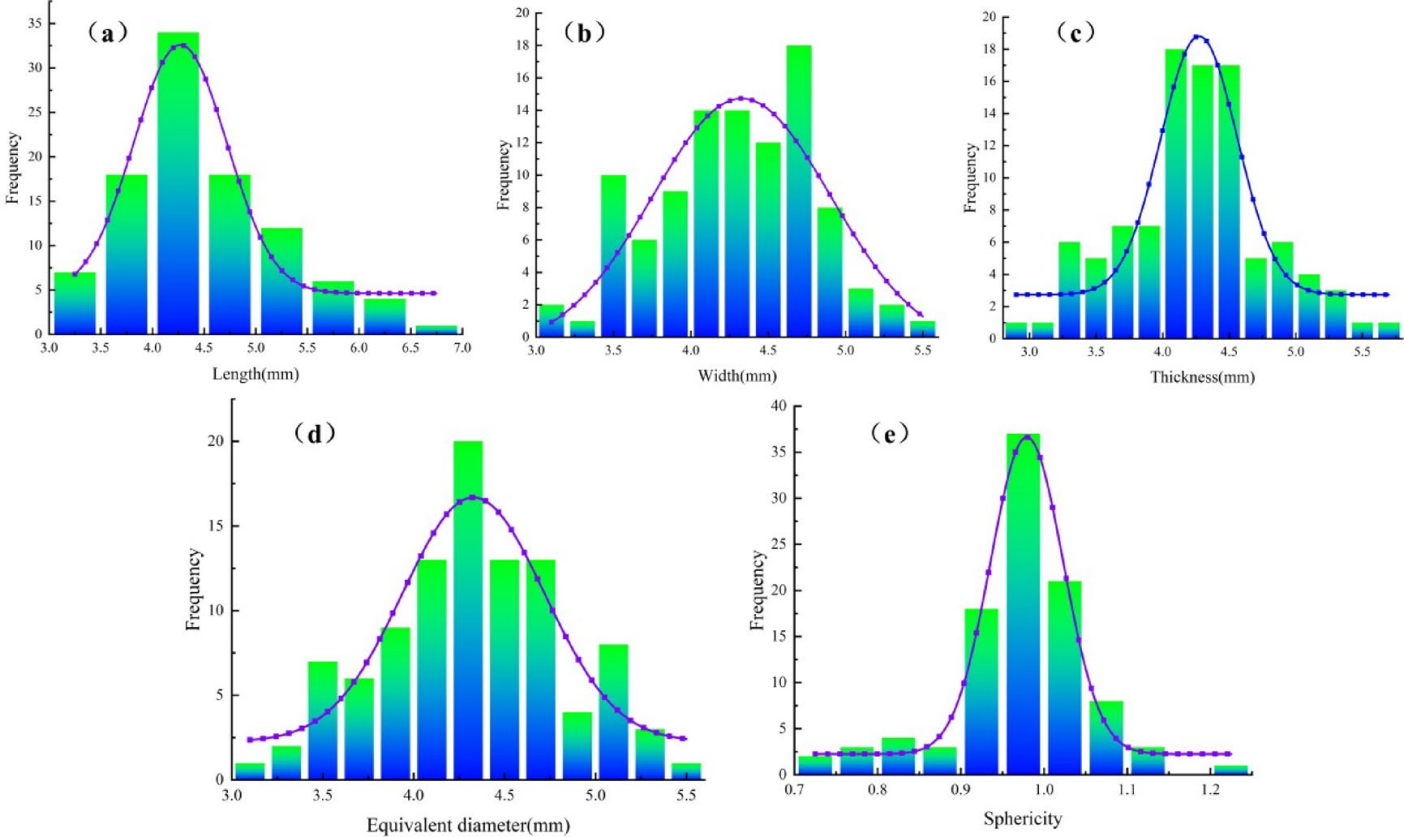
Histogram of the basic size distribution of the organic fertilizer particles. (**a**) The length distribution histogram; (**b**) The width distribution histogram; (**c**) The thickness distribution histo-gram; (**d**) The equivalent diameter distribution histogram; (**e**) The sphericity distribution histogram.

Following additional analysis and fitting using Origin 2019 software, the size distribution histogram is presented in Fig. 2. The length distribution of the organic fertilizer particles was approximately 82% within the range of 3.75 –5.25 mm. The width distribution ranged from around 94% within the 3.5 –5.1 mm interval. The thickness distribution ranged from around 87% within the 3.5 –5.1 mm interval. The corresponding diameter dispersion was around 93% within the 3.5 –5.1 mm range. The sphericity distribution was around 80% within the range of 0.9 – 1.1%.

#### The Poisson ratio of organic fertilizer particles

**Fig. 3:**
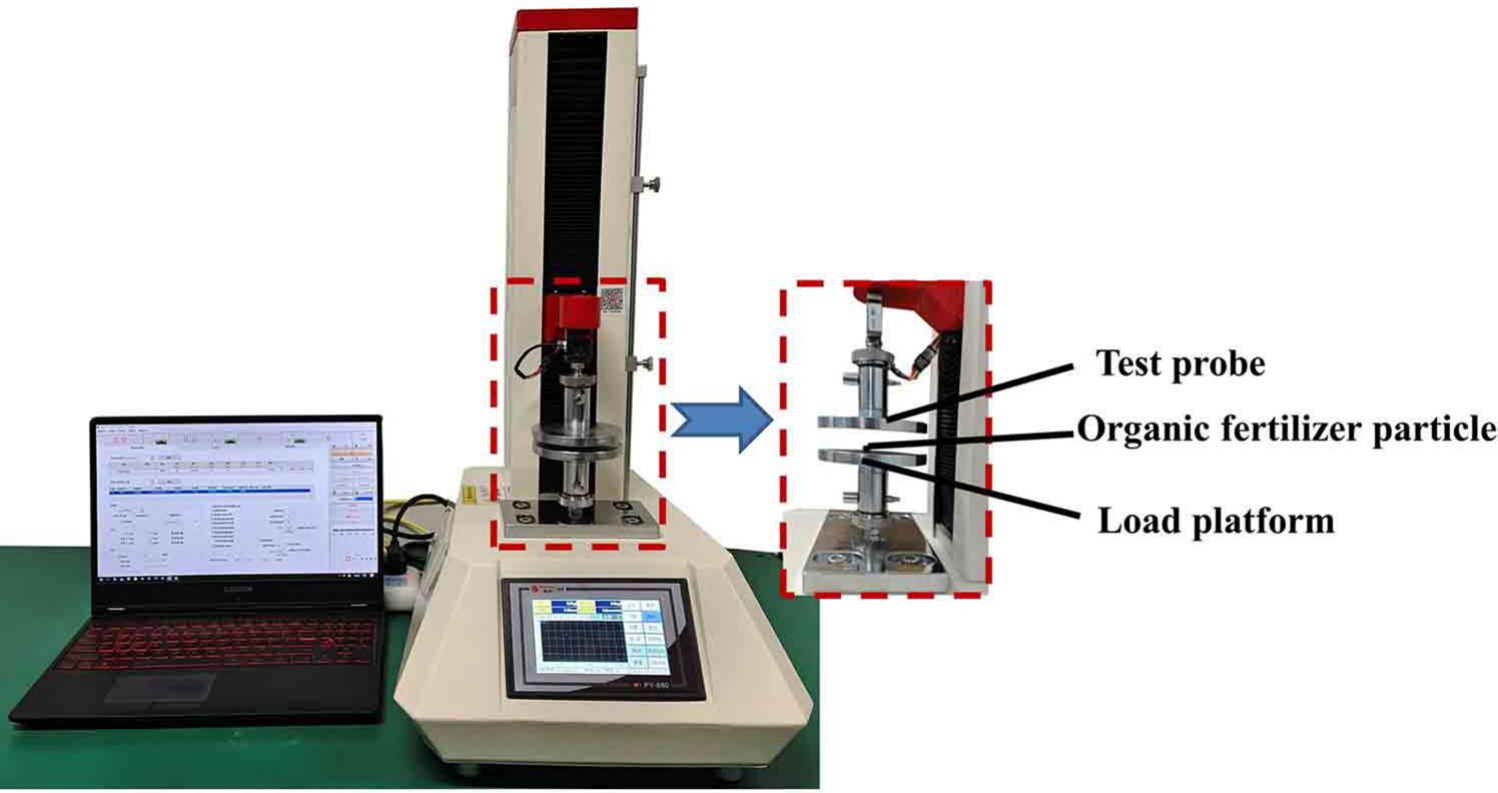
Poisson ratio determination test.

The ratio of horizontal strain to longitudinal strain of fertilizer particles during the extrusion process is the Poisson’s ratio of the fertilizer particles^26^. Figure 3 shows that the Poisson ratio of the organic fertilizer particles was determined using an electric tension and pressure testing device. The organic fertilizer particles were placed at the center of the loading platform, and their horizontal and longitudinal dimensions were measured. Loading was conducted via a test probe at a rate of 5 mm/min, with an initial force established at 0.981 N. Upon the rupture of organic fertilizer particles, slow down loading and measure the horizontal and longitudinal sizes of the fertilizer particles post-loading. The earlier tests were conducted ten times to obtain the average value, resulting in a computed Poisson ratio of 0.316 for the organic fertilizer particles, as per Formula (2). The elastic modulus is calculated to be 9.83 × 10^7^ Pa using Formula (3).

2 $$\mu =\frac{{{\varepsilon _L}}}{{{\varepsilon _d}}}=\frac{{{{\Delta L} \mathord{\left/ {\vphantom {{\Delta L} L}} \right. \kern-0pt} L}}}{{{{\Delta d} \mathord{\left/ {\vphantom {{\Delta d} d}} \right. \kern-0pt} d}}}$$

3 $$E=\frac{\sigma }{\varepsilon }$$

Where *µ* is Poisson ratio; *E* is the elastic modulus of organic fertilizer particles, Pa; $\sigma$ is stress, Pa; $\varepsilon$ is strain; ${\varepsilon _L}$ is the horizontal strain of the organic fertilizer particles; ${\varepsilon _d}$ is the longitudinal strain of the organic fertilizer particles; $\Delta L$ is the lateral deformation of organic fertilizer particles, mm; *L* is the original horizontal length of organic fertilizer particles, mm; $\Delta d$is the longitudinal deformation of organic fertilizer particles, mm; *d* is the original longitudinal length of organic fertilizer particles, mm.

#### Determination of the static friction coefficient

**Fig. 4:**
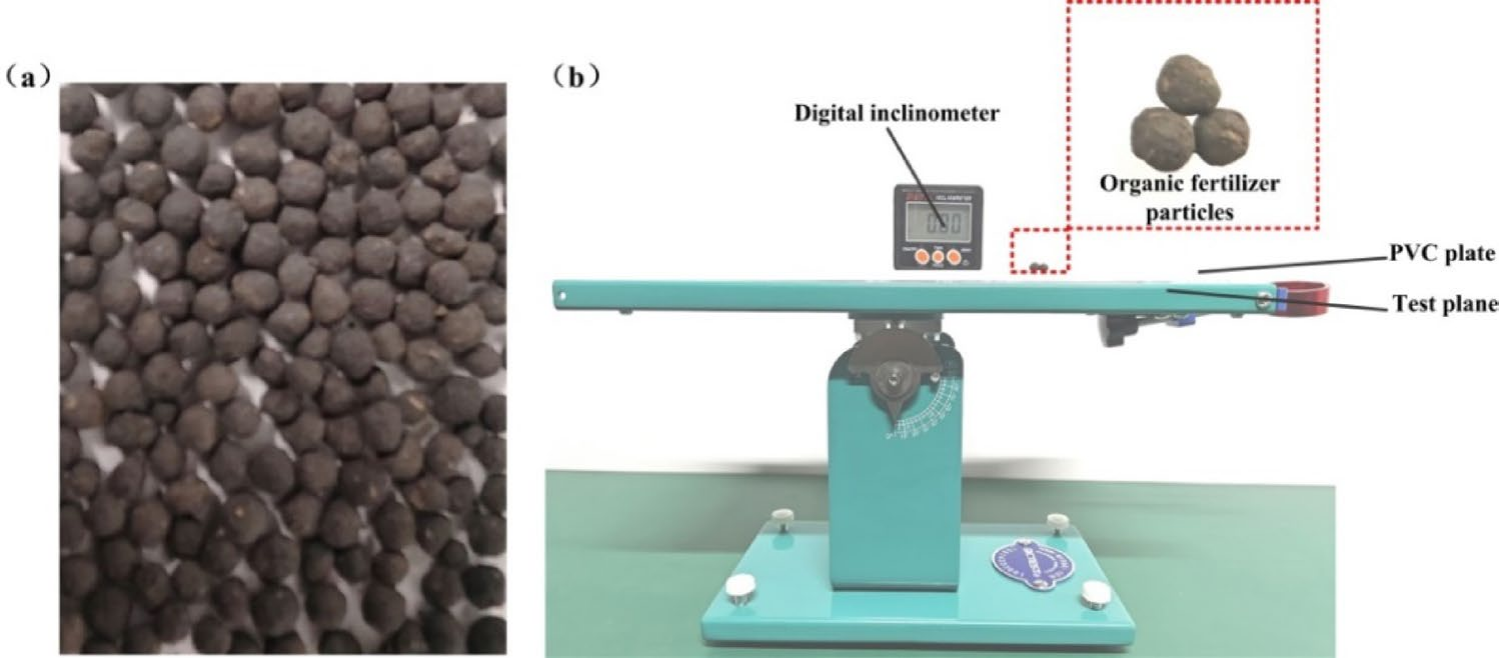
Static friction coefficient test. (**a**) The organic fertilizer particle plate. (**b**) Static friction coefficient test device.

During the fertilization process, organic fertilizer primarily interacts with essential components including the fertilizer box, distributor, and pipeline, with the friction coefficient being an essential characteristic. Figure 4 illustrates that three organic fertilizer particles were adhered together for the static friction test to reduce rolling and minimize testing errors. To determine the static friction coefficient between organic fertilizer particles, the particles were adhered to a particle plate. The PVC plate (organic fertilizer particle plate) was affixed to the test surface, and the angle of inclination was measured using a digital inclinometer. The rotation ceased when the attached organic fertilizer particles started to slide, and the inclination angle was documented at that moment. The coefficient of static friction was determined using Formula (4). The previous tests were carried out ten times to obtain average values. The coefficient for static friction between organic fertilizer particles and a PVC plate ranged from 0.27 to 0.33, while the coefficient of static friction between organic fertilizer particles themselves ranged from 0.46–0.52.

4 $${\mu _1}=\frac{{mg\sin {\alpha _1}}}{{mg\cos {\alpha _1}}}=\tan {\alpha _1}$$

Where ${\alpha _1}$ is the inclination angle of the test plane, ( °); *m* is the mass of organic fertilizer particles, g.

#### Determination of the rolling friction coefficient

The techniques and methodologies employed in assessing the rolling friction coefficient closely resemble those utilized in evaluating the static friction coefficient. Modify the inclination of the test plane of the inclinometer until the organic fertilizer particles commence rolling, and document the angle on the digital inclinometer. The rolling friction was determined using Formula (5). Each test group was conducted ten times, and the mean value constituted the final result. The coefficient for rolling friction between organic fertilizer particles and a PVC plate ranged from 0.16 to 0.20, whereas the coefficient between organic fertilizer particles was in 0.36–0.42.

5 $${\mu _2}=\tan {\alpha _2}$$

Where ${\mu _2}$ is the rolling friction coefficient. ${\alpha _2}$ is the critical angle of the rolling friction coefficient, ( °).

### Establishment of the organic fertilizer particles model

The discrete element approach has been widely used to investigate the movement and forces of bulk particles in crop products. The Bonding model simulates the bonding force between particles through a fixed size bond. The bond will break down under the influence of an external force, which is frequently used to replicate the phenomenon of crushing and breaking^27,28^. Consequently, the Bonding model was used to the organic fertilizer particle model. The Hertz − Mindlin with Bonding V2 contact model is an improvement of the original Bonding model. The complex particle function can facilitate the rapid formation of composite particles of varying sizes. The spherical units of the organic fertilizer particles are formed using bonding contacts. The bonding point is resistant to both tangential and normal displacements externally. The maximal normal and tangential shear stresses must be attained for adhesive breakdown.

**Fig. 5:**
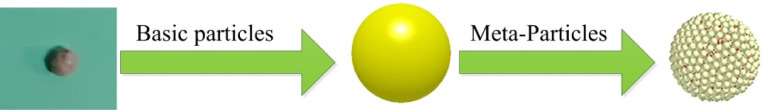
The organic fertilizer particle simulation model.

This study used a small quantity of particles to accurately illustrate the physical structure of regularly commanded organic fertilizer particles. The geometric model of the organic fertilizer particles was built using the Creo software, based on the particle size determined from physical testing. The organic fertilizer particle model was transformed into*.stp format and subsequently put into the discrete element simulation software. A specific quantity of spheres were created within the particle, and the spherical coordinate data among each particle was collected in the post-processing interface. After acquiring all particle coordinates, these were imported into the EDEM software. The particles were rapidly infused with meta-particles. Due to the smaller size of the organic fertilizer particles, they were collected into spherical particles with a diameter of 0.2 mm. The total quantity of particles was 673. Figure 5 shows the simulation model of the organic fertilizer particles.

### Repose angle test

**Fig. 6:**
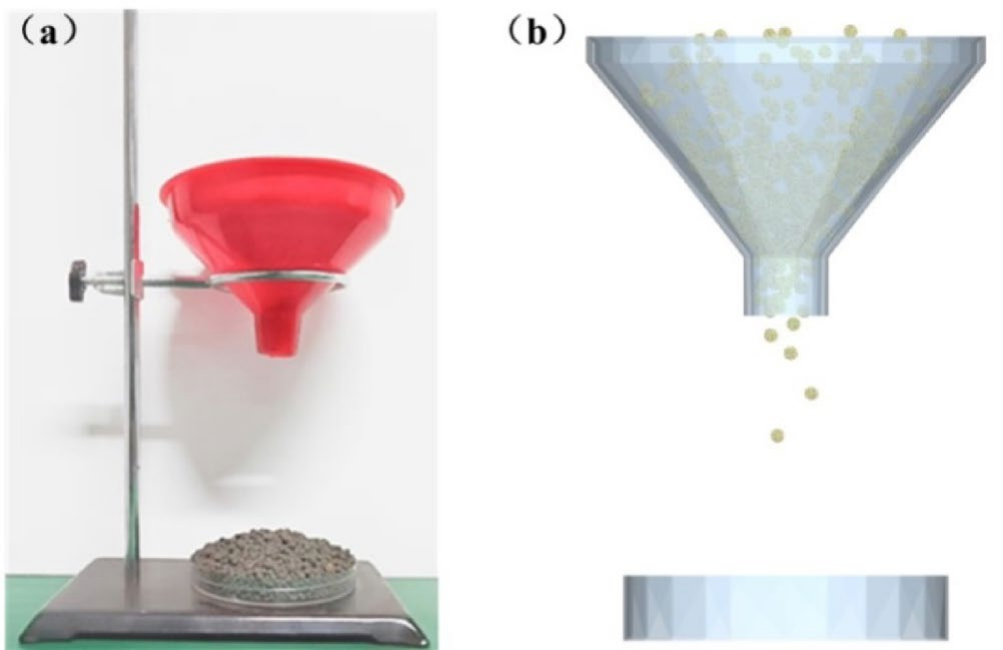
The repose angle test. (**a**) Physical tests. (**b**) Simulation tests.

The repose angle of the organic fertilizer particles was measured by the funnel method. The organic fertilizer particles were introduced by the hole above the funnel and allowed to slide down freely. When the organic fertilizer particles were totally still in the test box, the high-definition camera obtained the front view of the particles. The repose angle was calculated using Matlab 2022 to analyze the image of the organic fertilizer particle accumulation. The repose angle test can be seen in Fig. 6a. Each test group was conducted 10 times, and the average result formed the final value. The average repose angle of the organic fertilizer particles during the physical test was 18.15°.

Figure 6b shows that a particle factory was established above the funnel during the repose angle simulation. The organic fertilizer particles dropped under the effects of gravity and collected at the bottom of the test box. Upon support of the particle group, the angle represented the angle of repose between the inclined plane and the horizontal plane of the particle accumulation. The organic fertilizer particles were produced continuously. The generating rate was 100 g per second. The length of time of the simulation test was 2 s. The Rayleigh time step was established at 10%. The data stored was 0.01 s. The mesh size is three times the smallest particle radius.

### Parameter calibration test design of organic fertilizer particles

**Fig. 7:**
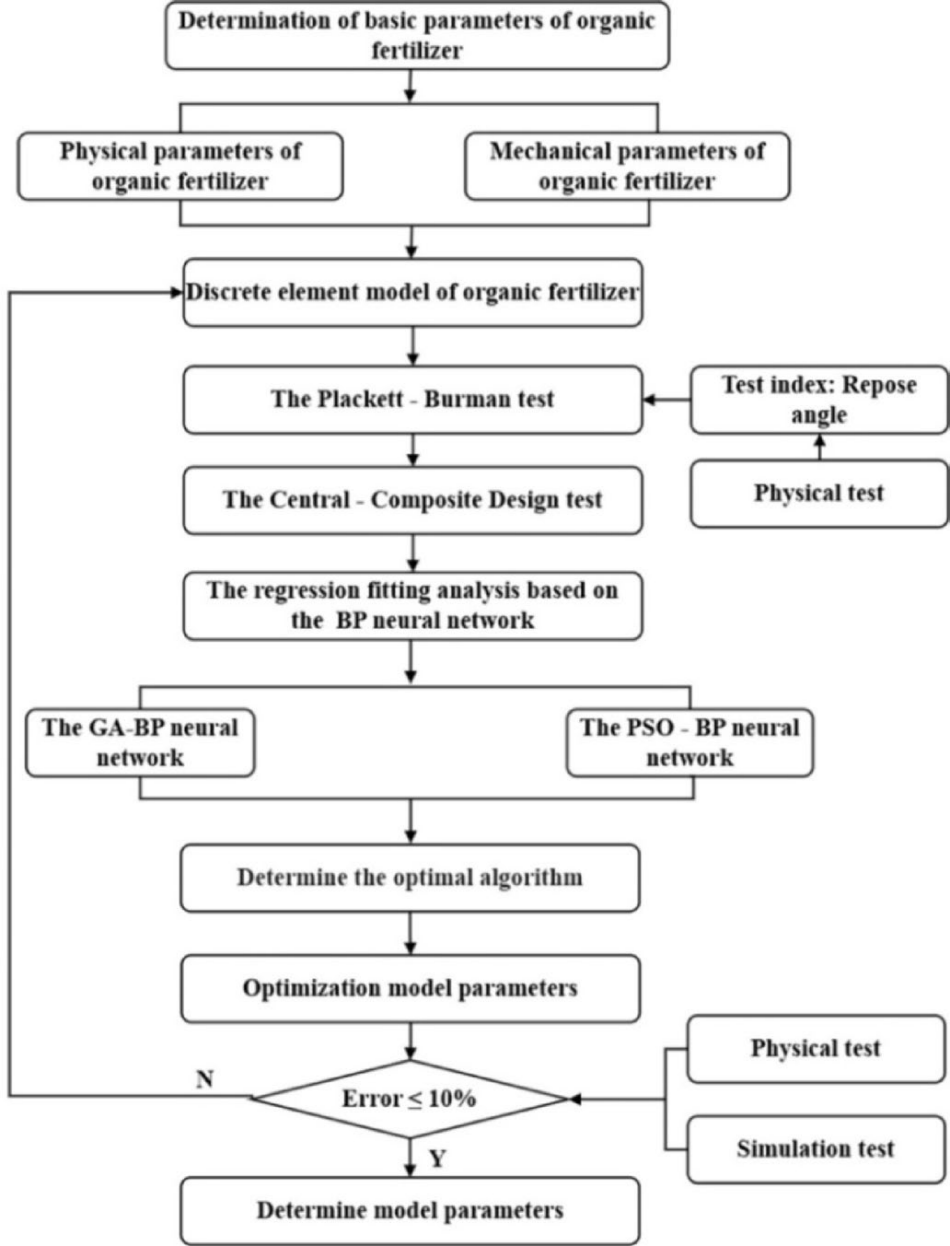
Experiment flowchart for calibrating simulation parameters of organic fertilizer particles.

The Plackett-Burman test was used for preliminary screening to rapidly determine the principal elements impacting the test signs. Central combination tests were used to improve the previously identified essential components. The regression fitting models of the BP neural network were created using the test results from the Central-Composite Design as the data set. The BP neural network was enhanced by the genetic algorithm (GA) and the particle swarm optimization method (PSO). The most optimal combination of simulation parameters for organic fertilizer particles was achieved. Figure 7 presents a flowchart of the study approach used.

#### The Plackett − Burman test

The parameters for the Plackett − Burman test were calculated from the measured results of physical tests, while further simulation parameters were referenced from important literature^29,30^. The repose angle had been chosen as the response variable, and the Plackett-Burman test was used to identify the parameters that significantly affect this response parameter. The minimum and maximum values of the test parameters were represented as − 1 and + 1, respectively, in Table 2. when each round of simulation testing, the repose angle of the organic fertilizer particles was determined.

**Table 2:** The Plackett − Burman test parameters.

NO.	Test parameters	Encoding		
		Low(-1)	Middle(0)	Hight(+ 1)
COR_O−O_	Recovery coefficient between organic fertilizer particles and organic fertilizer particles	0.2	0.4	0.6
COR_O−p_	Recovery coefficient between organic fertilizer particles and PVC plate	0.1	0.45	0.8
COS_O−O_	Static friction coefficient between organic fertilizer particles and organic fertilizer particles	0.46	0.49	0.52
COS_O−p_	Static friction coefficient between organic fertilizer particles and PVC plate	0.27	0.30	0.33
COD_O−O_	Rolling friction coefficient between organic fertilizer particles and organic fertilizer particles	0.36	0.39	0.42
COD_O−p_	Rolling friction coefficient between organic fertilizer particles and PVC plate	0.16	0.18	0.20
NSC_O−O_	Normal stiffness coefficient (MN/m^3^)	1	15.5	30
TSC_O−O_	Tangential stiffness coefficient (MN/m^3^)	5	12.5	20
NCS_O−O_	Normal critical stress (MPa)	0.04	0.07	0.1
TCS_O−O_	Tangential critical stress (MPa)	0.025	0.053	0.08

#### Central composite design test

The range of significant influencing factors was determined using the Plackett-Burman test. The average value of the relevant simulation parameters was chosen. The significant parameters served as test factors, while the repose angle used was the test index. Table 3 showed the horizontal coding of the repose angle simulation parameters.

**Table 3:** Horizontal coding table of the parameters.

Levels	Test parameters			
	COR_O−*p*_	COS_O−O_	COS_O−*p*_	COD_O−O_
−2	0.1	0.46	0.27	0.36
−1	0.28	0.48	0.29	0.38
0	0.45	0.49	0.3	0.39
+ 1	0.63	0.51	0.32	0.41
+ 2	0.8	0.52	0.33	0.42

### The regression fitting analysis based on the machine learning algorithm

#### Neural network construction and training

**Fig. 8:**
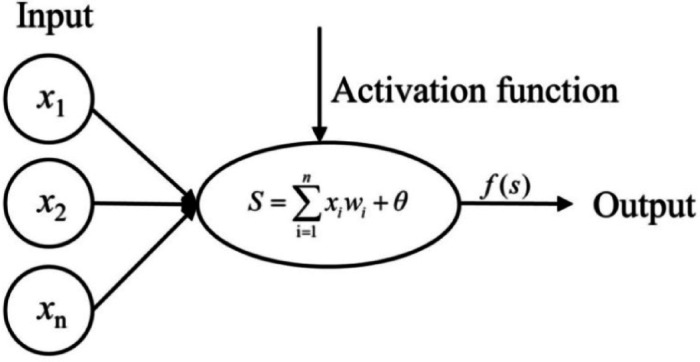
Basic structure of the neurons^31^.

The neural network model was a series of interconnected neuron layers, mainly based on three layers: the input layer, the hidden layer, and the output layer. The number of nodes in the input layer was calculated by the number of input parameters. The size of nodes in the output level was set through a set of output parameters. Each layer was linked by neurons. These neurons transmitted information between layers. The information was sent to the output layer in this manner. The changes in neuronal states can influence the correlation between input and output. Figure 8 shows the basic framework of a neuron.

Where *x*_1_、*x*_2_, ⋯*x*_n_ is the input training samples of neurons, *w*_1_、*w*_2_, ⋯*w*_n_ is the weight of the input node connected to the neuron node. *n* is the number of input neurons in the previous layer network. $\theta$ is the biased term of the neuron, then the weighted input S of the neuron node is:

6 $${\text{ne}}{{\text{t}}_i}=\sum\limits_{{i=1}}^{n} {{x_i}{\omega _i} - {\theta _i}}$$

The state output of this neuron is:

7 $${y_i}=f({\text{ne}}{{\text{t}}_i})$$

Where $f(\cdot )$ is the activation function or transfer function, which establishes a nonlinear transformation relationship between the input and output of a neural network. ${\text{net}}$ is known as net activation.

The regression fitting models of BP, GA-BP, and PSO-BP were tested using the test results from the Central Composite Design as the dataset. Due to the small amount of data in this paper, the data sets, totaling 30 groups, were broken down into a training group of 24 groups (80%) and a validation group of 6 groups (20%)^32^. Table 4 shows the experimental design and results of the Central Composite Design.

**Table 4:** The experimental design and results.

No.	Parameters				Repose angleθ/(°)
	COR_O−*p*_	COS_O−O_	COS_O−*p*_	COD_O−O_	
1	0	0	2	0	22.71
2	−1	1	−1	−1	16.05
3	−1	1	−1	1	24.72
4	−1	−1	−1	−1	17.71
5	1	1	1	1	26.86
6	−1	1	1	−1	19.88
7	0	0	0	0	21.81
8	1	1	−1	1	23.41
9	0	0	0	0	17.79
10	1	−1	1	1	21.43
11	0	0	0	0	22.98
12	−2	0	0	0	19.53
13	1	−1	1	−1	18.04
14	0	2	0	0	25.66
15	−1	−1	−1	1	17.55
16	2	0	0	0	21.78
17	−1	−1	1	1	28.81
18	0	0	0	0	19.48
19	−1	1	1	1	35.15
20	0	0	−2	0	21.09
21	1	−1	−1	−1	28.24
22	1	1	1	−1	20.76
23	1	1	−1	−1	24.64
24	0	0	0	2	23.67
25	1	−1	−1	1	21.44
26	0	0	0	0	19.38
27	0	0	0	−2	15.84
28	0	0	0	0	20.08
29	0	−2	0	0	22.53
30	−1	−1	1	−1	17.01

#### The BP neural network

**Fig. 9:**
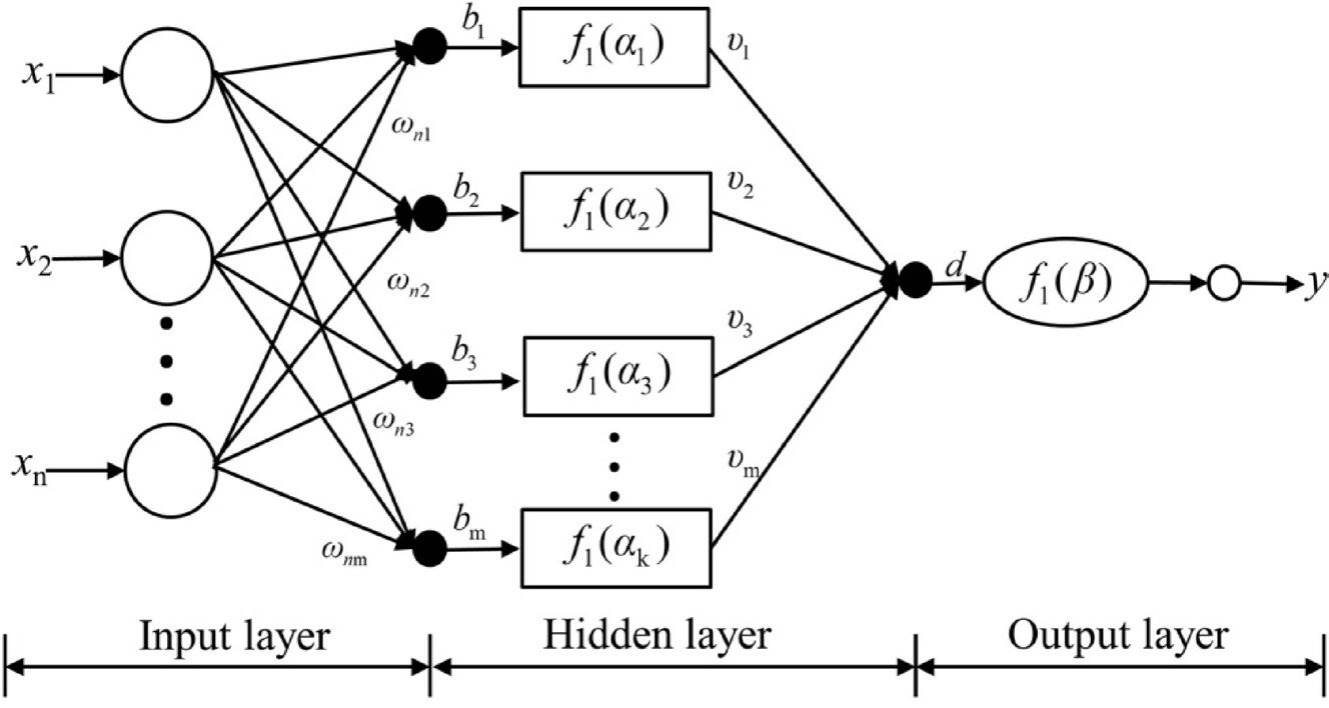
The BP neural network topology.

The BP neural network is a kind of multi-layer feedforward network that includes error backpropagation for training. The structure is quite simple, but it has significant self-learning and curve-fitting capacities. It is frequently employed in function fitting, optimization calculation, pattern recognition, and ideal prediction, among other applications^33,34^. The basic structure of the BP neural network can be seen in Fig. 9. *x* and y are the inputs and outputs of the model, respectively. *b* and d are the thresholds of the model. *w* and u are the weights of the model. *f*_1_ and *f*_2_ are the transfer functions of the model. The specific calculation steps of model training are as follows:

The weights and thresholds between node *j* of the hidden layer and node *k* of the output layer are set as *u*_jk_ and *d*_k_, respectively. Then the output value *y*_k_ of node *k* in the output layer is:

8 $${y_k}={f_2}(\sum\limits_{{j=1}}^{m} {{u_{jk}}{h_j}+{d_k}} )$$

Set *t*_k_ as the expected output value of the sample, then the error of this training is:

9 $$E=\frac{1}{2}\sum\limits_{{k=1}}^{p} {({y_k} - {t_k}} {)^2}$$

The training error *E* is used as the basis for the adjustment of model parameters in the process of backpropagation. The weight and threshold adjustments are ∆*u*_jk_(*t*) and ∆*d*_k_(*t*), respectively. In the *t* + 1 iteration, the weight and threshold adjustment formula are as follows:

10 $${u_{jk}}(t+1)={u_{jk}}(t)+\Delta {u_{jk}}$$

11 $${d_k}(t+1)={d_k}(t)+\Delta {d_k}$$

The training group samples are analyzed iteratively until an error between the predicted value and the expected value serves the standards. This study utilized a single hidden layer construction, applying COR_O−p_, COS_O−O_, COS_O−p_ and COD_O−O_ as input layers. The repose angle created the output layer. The hidden layer has been set to 9 based on the relevant research^35,36^. Therefore, the BP neural network model had a structure with three layers with a structure of 4-9-1. The learning algorithm employed the Levenberg-Marquardt algorithm, and the mapminmax function was utilized to normalize the input and output data, so reducing the impact of dimensionality. A total of 80 iterations, the target error was set at 0.001 during training, and the learning rate was set at 0.001.

#### The GA − BP neural network

The GA-BP neural network is a hybrid method which uses the genetic algorithm with the error backpropagation algorithm to train feedforward artificial neural networks. The rate of network convergence increases, and local minimization is avoided. This network converges quickly that reaches the optimal solution with simple. The genetic algorithm facilitates the evolution of each individual towards enhanced fitness via selection, crossover, mutation, and additional processes. The GA − BP established a population size of 150 and a total of 280 iterations. The cross coefficient was 0.03. The function of the coefficient of variation was the nonUnifMutation.

#### The PSO − BP neural network

In the PSO-BP model, Particle Swarm Optimization (PSO) is used to determine globally optimal initially weights and thresholds for Backpropagation (BP) neural networks. Particle Swarm Optimization (PSO) models include the global particle swarm model and the local particle swarm model. It is a global model where particles check both individual and collective extrema during iterative movement. This paper employed the PSO global model for optimization. The PSO algorithm was used to determine the optimal weights and thresholds of the BP neural network before, therefore improving the convergence rate and enhancing the learning efficiency of the network. The PSO algorithm depends on collaboration and information interaction among others in the group, with each particle independently searching its own search space for the optimal solution and sharing ideas with other particles to determine the current global optimal. Each particle changed its velocity and location to correspond to the currently global optimal solution, continuously repeating to improve this solution. The PSO-BP model set the maximum iteration count at 68 and the population size at 38. The individual learning factor is -5 − 5, the social learning factor is -5–5, and the inertia weight is 2.

#### The data analysis and processing

This study used the Matlab R2022b software to set up machine learning methods. The predictive effectiveness of the machine learning models was evaluated by R^2^MAE, and RMSE variables^37^.

## Results

### The Plackett − Burman test results analysis

According to the several variables that affect in the repose angle test between organic fertilizer particles and the PVC plate, a Plackett-Burman test is needed to determine the importance of each factor’s influence. The contact parameters between organic fertilizer particles and the PVC plate were evaluated by the repose angle as the response factor. A total of 12 test groups were accomplished. Each test group was tested three times, and the average value was kept as the final results. The method of testing and results are given in Table 5. The significance analysis of the parameters using the Plackett-Burman test is given in Table 6. Through significance analysis, it was found that COR_O−p_, COS_O−O_, COS_O−p_ and COD_O−O_ had significant effects on the repose angle of the organic fertilizer.

**Table 5:** The Plackett − Burman test scheme and results.

No.	Parameters											Repose angle θ/(°)
	COR_O−O_	COR_O−*p*_	COS_O−O_	COS_O−*p*_	COD_O−O_	COD_O−*p*_	NSC_O−O_	TSC_O−O_	NCS_O−O_	TCS_O−O_		
1	1	1	1	−1	−1	−1	1	−1	1	1		13.66
2	−1	1	1	1	−1	−1	−1	1	−1	1		16.64
3	−1	−1	−1	1	−1	1	1	−1	1	1		25.67
4	−1	1	−1	1	1	−1	1	1	1	−1		33.56
5	1	−1	−1	−1	1	−1	1	1	−1	1		17.68
6	1	1	−1	1	1	1	−1	−1	−1	1		36.52
7	−1	−1	−1	−1	−1	−1	−1	−1	−1	−1		9.56
8	1	1	−1	−1	−1	1	−1	1	1	−1		14.53
9	−1	−1	1	−1	1	1	−1	1	1	1		12.86
10	1	−1	1	1	1	−1	−1	−1	1	−1		15.47
11	−1	1	1	−1	1	1	1	−1	−1	−1		20.89
12	1	−1	1	1	−1	1	1	1	−1	−1		13.46

**Table 6:** Significance analysis of the Plackett − Burman test parameters.

Parameters	Stdized effects	Sum of squares	Contribution (%)	Significance order
COR_O−O_	−1.31	5.15	0.65	9
COR_O−p_	6.85	140.77	17.77	4
COS_O−O_	−7.42	165.32	20.87	2
COS_O−p_	8.69	226.55	28.60	1
COD_O−O_	7.24	157.40	19.97	3
COD_O−p_	2.89	25.11	3.17	6
NSC_O−O_	3.22	31.17	3.93	5
TSC_O−O_	−2.17	14.17	1.79	8
NCS_O−O_	0.17	0.083	0.011	10
TCS_O−O_	2.59	20.18	2.55	7

### Analysis of the central − composite design test results

The results of variance analysis of the Central Composite Design test are shown in Table 7. It can be seen that COD_O−O_, COR_O−p_COS_O−p_, COR_O−p_COD_O−O_, COS_O−O_COD_O−O_, COS_O−p_COD_O−O_ and COS_O−O_^2^ had extremely significant effects on the relative error of the repose angle. The COS_O−O_, COS_O−p_ and COS_O−O_COS_O−p_ had significant effects on the relative error. The repose angle fitted to the regression model *P* < 0.0001, which means an important relationship between the repose angle and the regression equation. The loss of fit term *P* = 0.8433 > 0.05 indicate that the model fit well. The no-loss phenomenon occurred. The coefficient of determination of the regression equation was *R*² = 0.9337, showing a value quite similar to 1. The regression model was of great importance and can accurately represent the actual conditions. It can be used for accurate analysis of the target repose angle.

**Table 7:** Variation analysis of central composite design test quadratic model.

Source of variance	Mean square	Degree of freedom	Sum of square	*P*-value
Model	481.83	14	34.42	< 0.0001**
COR_O−p_	6.45	1	6.45	0.1135
COS_O−O_	31.51	1	31.51	0.0021*
COS_O−p_	12.64	1	12.64	0.0326*
COD_O−O_	115.72	1	115.72	< 0.0001**
COR_O−p_COS_O−O_	4.2	1	4.2	0.1948
COR_O−p_COS_O−p_	78.59	1	78.59	< 0.0001**
COR_O−p_COD_O−O_	72.76	1	72.76	< 0.0001**
COS_O−O_COS_O−p_	11.36	1	11.36	0.0414*
COS_O−O_COD_O−O_	26.47	1	26.47	0.0039**
COS_O−p_COD_O−O_	81.36	1	81.36	< 0.0001**
COR_O−p_^2^	1.97	1	1.97	0.3677
COS_O−O_^2^	34.89	1	34.89	0.0014**
COS_O−p_^2^	9.2	1	9.2	0.063
COD_O−O_^2^	0.051	1	0.051	0.8837
Residual	34.23	15	2.28	
Lack of fit	16.91	10	1.69	0.8433
Pure error	17.32	5	3.46	
Cor total	516.06	29		

** in Table 7 indicates that the impact is extremely significant (*P* < 0.01), and * indicates that the impact is significant (*P* < 0.05).

### Regression fitting analysis of the machine learning

#### Model comparative analysis

The regression fitting model of the data was fitted by four algorithms, and the R², MAE, and RMSE of the model were compared to determine the most suitable regression model for the calibration of organic fertilizer particle simulation parameters. Table 8 shows the model comparison results of the four algorithms that follow repeated training. The comparing findings of the four models showed that R^2^ had been ordered below: PSO − BP > GA − BP > BP > RSM. The GA-BP combined a genetic algorithm into a BP neural network, the PSO − BP merged a particle swarm optimization approach with a BP neural network, and the BP represented a conventional error backpropagation method. The algorithm search performance of the BP neural network causes it to settle on local ideal solutions, which can limit its overall effectiveness in finding the global optimum. The PSO algorithm’s global search ability significantly reduced the problem and significantly improved the model’s predictive accuracy. The GA − BP built the global searches of the genetic algorithm with the local search abilities of the BP algorithm. The MAE comparison results of the four algorithms were the following: PSO − BP > GA − BP > BP > RSM. The RMSE comparison generated a set of results: PSO − BP > GA − BP > BP > RSM. The PSO − BP blended particle swarm optimization approach and the BP neural network has left the ability of finding global optimal approaches and performing carefully local optimization, which contributed for its better overall success. The results matched with the findings of Huang (2021) and Mao (2022), led to the choice of the PSO − BP algorithm for further study^38,39^. It is great in model accuracy, stability, and fitting. This work follows the PSO − BP method to attain better fitting results, which allows the buildup of a model with enhanced accuracy and reduced mistakes.

**Table 8:** The model comparison.

Algorithm	BP	GA − BP	PSO − BP	RSM
R^2^	0.9204	0.9683	0.9758	0.9337
MAE	0.4691	0.3998	0.3631	1.2456
RMSE	0.3590	0.3173	0.282	0.5384

#### Model evaluation

**Fig. 10:**
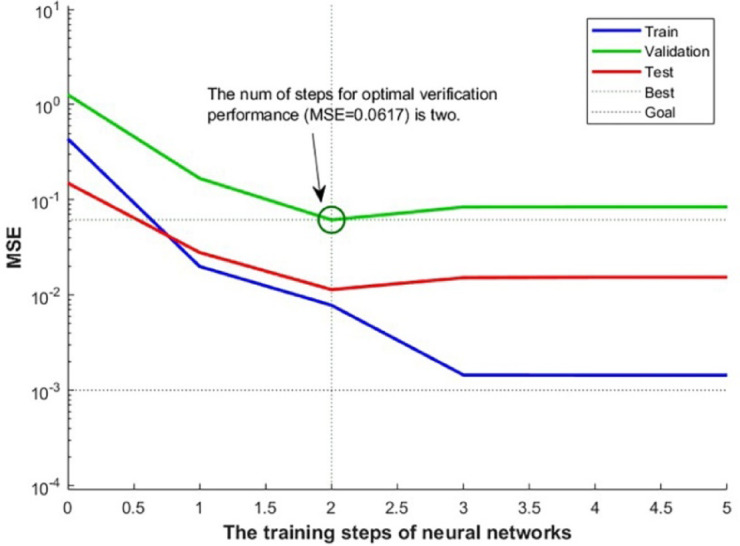
The MSE performance evaluation.

Figure 10 shows the MSE performance evaluation of the PSO − BP model. The model’s MSE showed a decrease during the training stage, indicating an improvement in its match to the training data with period. The optimal performance was achieved at the initial stage of training, when the neural network’s training was basically finished. The results showed that the PSO − BP training convergence was efficient and stable, enabling the model suited to test purposes.

**Fig. 11:**
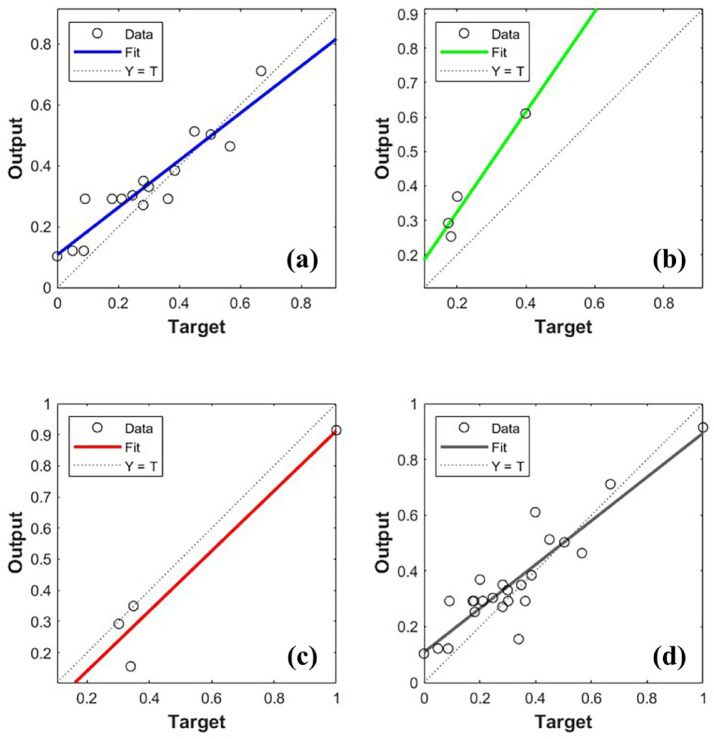
Regression analysis. (**a**) Training set, (**b**) Validation set, (**c**) Testing set, (**d**) Whole data set.

Figure 11 shows the training, verification, and testing performances of the PSO − BP algorithm model. The correlation coefficients of training, verification, testing, and the whole data set were 0.9317, 0.9734, 0.9575, and 0.9034, respectively, showing the accurate fitting effectiveness of the model. The correlation coefficients of the data were accurate, showing a lack of significant overfitting or underfitting. The PSO − BP method showed outstanding efficacy, creating a model characterized by high accuracy and robust generalization abilities in this study. The model works to further tests.

#### Optimization test of the PSO − BP algorithm

The model created by the PSO − BP method shows better fitting accuracy. The PSO − BP method was used to iterate until someone with the maximal fitness was achieved. The best parameter combinations the following: COR_O−p_ was 0.35, COS_O−O_ was 0.49, COS_O−p_ was 0.29, and COD_O−O_ was 0.38. The repose angle of the organic fertilizer particles was simulated by the optimal parameter combination of the PSO − BP algorithm. The repose angle measured 17.87°, with an error margin of 1.54% relative to the physical test. The particle model and characteristics of organic fertilizer established at the research structure may acts as a basis for future creation of an original channel-wheel fertilizer discharge apparatus.

## Discussion

The study uses a broad approach combining physical testing, simulations, and machine vision techniques to calibrate the parameters of organic fertilizer particles. The fundamental physical property characteristics of organic fertilizer particles were determined through physical testing. The Plackett-Burman test was used for initial analysis. The parameters that greatly determine the repose angle are identified. The Central-Composite Design test was used to improve the previously identified key elements. The regression fitting models of the BP neural network have been developed using the test results from the Central Composite Design as the dataset. The BP neural network was improved through the use of genetic algorithms (GA) and particle swarm optimization (PSO). The R^2^MAE, and RMSE of the BP, GA − BP, PSO − BP, and RSM regression models were compared and evaluated. The results showed that the PSO − BP algorithm could get better fitting efficiency and develop a prediction model with better precision and reduced error for analyzing the repose angle of organic fertilizer particles. The PSO − BP method was employed to iterate until the person with the optimal fitness was achieved. The best parameter combinations the following: COR_O−p_ was 0.35, COS_O−O_ was 0.49, COS_O−p_ was 0.29, and COD_O−O_ was 0.38.

This paper took the reliable sheep manure organic fertilizer made by Inner Mongolia Pure Sheep Effective Organic Fertilizer Limited Liability Company. It will analyze the algorithms limited extrapolation capability to enhance its accuracy and achieve the ideal parameter configuration. We want to persistently gather additional sample data during the forthcoming years and improve the experimental design to progressively improve the diversity and representativeness of the data. This entails broadening the parameter range and integrating supplementary environmental parameters to enhance its relevance and precision across diverse planting situations.

## Conclusion

This study takes a comprehensive approach which includes simulations, machine vision techniques, and physical tests to calibrate the organic fertilizer particles. The regression fitting models of the BP neural network were created using the test results from the Central − Composite Design as the data set. The BP neural network was enhanced by the genetic algorithm (GA) and the particle swarm optimization method (PSO). The ideal combination of simulation parameters for organic fertilizer particles was achieved. The most important conclusions are as followed.

1. The fundamental physical property characteristics of organic fertilizer particles were determined through physical examination. The Poisson ratio of organic fertilizer particles was 0.316, and the elastic modulus was 9.83 × 10^7^ Pa. The COS_O−p_ ranged from 0.27 to 0.33. The COS_O−O_ ranged from 0.46 to 0.52. COD_O−p_ ranged from 0.16 to 0.20. The COD_O−O_ range was 0.36–0.42.
2. The Plackett-Burman test was used for initial analysis. The parameters that greatly determine the repose angle are identified. The Central-Composite Design test was used to improve the previously identified key elements. The regression fitting models of the BP neural network have been developed using the test results from the Central Composite Design as the dataset. The BP neural network was improved through the use of genetic algorithms (GA) and particle swarm optimization (PSO).
3. The R^2^MAE and RMSE of the BP, GA − BP, PSO − BP and RSM regression models were compared and analyzed. The results showed that PSO − BP algorithm could achieve better fitting effect, and could construct a prediction model with higher accuracy and less error to analyze the repose angle of the organic fertilizer particles. The PSO − BP algorithm was used to iterate until the individual with the closest fitness was obtained. COR_O−p_ was 0.35, COS_O−O_ was 0.49, COS_O−p_ was 0.29 and COD_O−O_ was 0.38 were the optimal parameter combination.

Due to the small amount of data in this paper, the data sets, totaling 30 groups, were broken down into a training group of 24 groups (80%) and a validation group of 6 groups (20%). We will investigate strategies to enhance the model architecture for limited sample datasets. We are contemplating the use of lightweight convolutional neural networks (CNNs) or pre-trained models leveraging transfer learning, as both methodologies can sustain robust predicting performance despite constrained training data.

## Data Availability

The original contributions presented in the study are included in the article, further inquiries can be directed to the corresponding author.
